# 

**Published:** 1914-06-15

**Authors:** 


### ANNOUNCEMENTS.

### The Panama-Pacific Dental Congress.

The Committee of Organization of the Panama-Pacific Dental Congress is pleased to present to the members of the dental profession the following report showing the progress of the work of organization, feeling certain that a sound foundation has been laid for the greatest dental meeting ever attempted.

Letters received by the committee from every state and country in the world indicate a widespread and lively interest in the congress and give promise of a record breaking attendance. At the present time, sixteen months before the opening of the congress, over one-third of the space for exhibits has been reserved, many applications are pending, and without doubt the whole twenty-two hundred front feet of exhibit space will soon be taken. Contributions to the program are now being received and the success of the meeting is assured. The Panama-Pacific International Exposition, as far as its buildings and grounds are concerned, is rapidly assuming a finished appearance. All the great exhibit palaces, eleven in number, will be ready for occupancy on or before the first day of next July, two of them being ready for exhibits at the present time.

An idea of the size of these buildings may be obtained by noting the fact that the machinery building, now finished, is one and one-third miles around the cornice and contains over nine million feet of lumber.

The Exposition will be "Ready on Time," and everyone should plan to see, what, for many years to come, will be the last word in great educational and industrial expositions.

The Dental Congress will be held during the pleasantest season of the year, when the Exposition will be at the height of its activity and no member of the dental profession, who can by any possibility come to San Francisco at that time, can afford to miss the Exposition and the Congress.

OFFICERS OF THE COMMITTEE OF ORGANIZATION.

Dr. Frank L. Platt, Chairman, 323 Geary street, San Francisco.

Dr. Arthur M. Flood, Secretary, 240 Stockton street, San Francisco.

OFFICERS OF THE SECTIONS OF THE PANAMA-PACIFIC DENTAL CONGRESS

*Section I.—Anatomy, Physiology and Histology.*

Dr. I. Norman Broomell, Chairman, Medico-Chirurgical College, Philadelphia, Pa.

Dr. Malcolm Goddard, Secretary, Butler Building, San Francisco, Cal.

*Section II.—Etiology, Radiography, Pathology and Bacteriology.*

Dr. Frederick B. Noyes, Chairman, 122 South Michigan avenue, Chicago, Ills.

Dr. W. H. Renwick, Secretary, Sacramento, Cal.

*Section III.—Chemistry and Metallurgy.*

Dr. M. L. Ward, Chairman, Ann Arbor, Mich.

Dr. H. A. Tuckey, Secretary, Head Building, San Francisco.

*Section IV.—Oral Hygiene and Prophylaxis.*

Dr. Herbert L. Wheeler, Chairman, 560 Fifth avenue, New York City, N. Y.

Dr. Robert W. Hall, Secretary, Salt Lake City, Utah.

*Section V.—Materia Medica and Therapeutics.*

Dr. J. P. Buckley, Chairman, 39 South State street, Chicago, Ills.

Dr. Frank C. Pearn, Secretary, North Pacific College of Dentistry, Portland, Ore.

*Section VI.—Oral Surgery.*

Dr. Truman W. Brophy, Chairman, 81 East Madison street, Chicago, Ills.

Dr. E. S. Barnes, Secretary, Cobb Building, Seattle, Wash.

*Section VII.—Orthodontia.*

Dr. J. Lowe Young, Chairman, 576 Fifth avenue, New York, City, N. Y.

Dr. James David McCoy, Secretary, Brockman Bldg., Los Angeles, Cal.

*Section VIII.—Operative Dentistry.*

Dr. John Sayre Marshall, Chairman, 2912 Pine avenue, Berkeley, Cal.

Dr. E. A. Tripp, Secretary, Salt Lake City, Utah.

*Section IX.—Prosthesis.*

Dr. Ellison Hillyer, Chairman, 1143 Dean street, Brooklyn, N. Y.

Dr. C. O. Edwards, Secretary, 3989 Howe street, Oakland, Cal.

*Section X.—Education, Nomenclature, Literature, History, Legislation.*

Dr. C. N. Johnson, Chairman, 22 East Washington street, Chicago, Ills.

Dr. Henry C. Fixott, Secretary, Portland, Ore.

RULES GOVERNING OFFICERS OF SECTIONS, AND STATE AND NATIONAL EXECUTIVE COMMITTEES.

Rules governing the Officers of Sections and Chairmen and Members of State and National Executive Committees of the Panama-Pacific Dental Congress, to be held in San Francisco, California, August 30 to September 9, 1915.

*Rule I.*—The Officers of each Section shall constitute the Board of Censors for that Section.

*Rule II.*—The Officers of each Section shall co-operate with State and National Executive Committees in securing papers and clinics for the program of the Congress, and also with the Program and Clinic Committees.

*Rule III.*—The Officers of each Section and the chairmen and members of State and National Executive Committees are empowered to solicit and receive from legal and reputable practitioners of dentistry and medicine, and persons proficient in the allied sciences, papers and clinics on subjects of interest to the Congress, it being understood that each essayist or clinician is an authority on, or particularly well qualified to deal with, the subject presented.

*Rule IV.*—The Chairman of each Section is invited to deliver an address before his Section, not to exceed twenty minutes in length; this address to constitute one of the papers of that Section.

*Rule V.*—The aggregate number of papers accepted shall not exceed ten for each Section, and not more than two-fifths of those accepted may be read by title.

*Rule VI.*—Papers may be read and discussed before the Congress in any language, but copies of all papers, or summaries of papers, and discussions, typewritten in the English language, ready for printing, must reach the Program Committee in San Francisco not later than May 1, 1915.

*Rule VII.*—Each paper and discussion will be printed in full in the published transactions of the Congress, but a maximum of twenty minutes only will be allowed for the reading of a paper, or a summary of it, embracing its leading points in case the reading of the original would occupy more than the allotted time, and five minutes for each speaker taking part in the discussion; not more than fifteen minutes will be allowed for the discussion of any paper, and the author will be allowed five minutes in closing the discussion.

The author of each paper is requested to furnish the Secretary of the Section to which his paper belongs with the names and addresses of those who will discuss his paper.

*Rule VIII.*—No clinic will be given a place on the program of the Congress unless a concise description of it, typewritten in the English language, ready for printing, reaches the Clinic Committee in San Francisco on or before May 1, 1915.

*Rule IX.*—State and National Executive Committees are governed by the rules governing the Officers of Sections, fo far as they apply. Note particularly Rules II, III, V, VI, VII and VIII; also

*Rule X.*—Each contribution to the program, either paper or clinic, shall be sent promptly to the Chairman of the Section in which its title indicates it to belong. In case of doubt, it shall be sent to the office of the Committee of Organization in San Francisco, this committee determining its place on the program.

*Rule XI.*—In the event of any controversy arising between contributors and the officers of any Section the question at issue shall, at the discretion of the officers of the Section, be submitted to the Committee of Organization for final adjustment.

### QUALIFICATIONS FOR MEMBERSHIP.

State and National Executive Committees are empowered to receive applications for membership from none but legal and reputable practitioners of dentistry, who are personally known to be such, vouched for by an officer of the principal dental society of their locality, or by some other known reputable and legal practitioner. Each application must be signed by a member of a State or National Executive Committee.

Membership fee is Ten Dollars.

Visitors to the Congress not eligible for membership.

Members may introduce members of their families as visitors to the Congress upon payment of a fee of $2.50.

### PROGRAM COMMITTEE.

Dr. E. E. Evans, Chairman, Union Savings Bank Building, Oakland, Cal.

### CLINIC COMMITTEE.

Dr. John D. Millikin, Chairman, 323 Geary street, San Francisco.

### ENTERTAINMENT COMMITTEE.

Dr. Frank C. Pague, San Francisco, Chairman; Dr. Paul A. Mariotte, Oakland; Dr. A. W. Ward, San Francisco; Dr. Edward Otis Whitney, San Francisco; Dr. Franklin H. Locke, Oakland.

### TRANSPORTATION COMMITTEE.

Dr. Henry Woods Weirick, Chairman, San Francisco; Dr. Harry R. Evans, New York City; Dr. Alpheus R. Brown, Boston, Mass.; Dr. E. M. Carson, St. Louis; Dr. F. W. Gethro, Chicago, Ills.; Dr. J. D. Eby, Atlanta, Ga.

---

### Tennessee State Dental Association.

The 47th annual meeting will be held June 25th to 27th, 1914, in Chattanooga. This is an historic place of much interest and as it is also centrally located a large attendance is expected. A good programme is in preparation and there are important business matters to be passed upon. All members should attend and all ethical dentists will be cordially welcomed.

C. O. Rhea, Secy.

---

### Xi Ipsi Phi Fraternity.

### NATIONAL ALUMNI ASSOCIATION ANNUAL MEETING.

"Good-fellowship, not Politics."

PLACE:—Rochester, N. Y.

DATE:—July 6th. All functions will be held on this day so as not to conflict with the sessions of the National Dental Association.

HEADQUARTERS:—Hotel Seneca. Register on arrival in the Xi Psi Phi Parlor on the Mezzanine. An entire floor has been reserved for our members.

FUNCTIONS:—Annual dinner in the large banquet hall of the hotel at 6 p. m. Members of international respect will do the toasting. Be sure to send your acceptance without further notice to Dr. George C. Lowe, 813 Chamber of Commerce Bldg., Rochester, N. Y., so that reservation may be made for you. This Is Important so that the correct number may be provided for.

The Annual Business Session will immediatly follow the banquet. Matters of the utmost importance will come up for disposal and your presence is, therefore, strongly urged.

MEMBERSHIP COMMITTEE:—Kindly get in touch with Dr. C. C. Markey, 1436 Peoples Gas Building, Chicago, Ills.

L. M. WAUGH, President, Buffalo, N. Y.

C. O. SIMPSON, Secretary, St. Louis, Mo.

### NATIONAL DENTAL ASSOCIATION.

### TENTATIVE PROGRAM.

The National Dental Association will hold its 1914 meeting in Rochester, N. Y., July 7th to 10th. The House of Delegates will hold its first session on Monday, July 6th, at 11 a. m., and it is important that all delegates be present at this time.

The first General Session will open at 11 a. m. Tuesday, July 7th, and the Local Committee have hopes that Gov. Glynn will be present to make the address of welcome. This will be responded to by Dr. B. Holly Smith, Baltimore, Md. The President's address will be followed by an address by Dr. Victor C. Vaughn, President of the American Medical Association.

The second General Session will be held in Convention Hall at 8 p. m. Tuesday and will be a symposium by the Research Commission with Drs. Weston A. Price, Thomas B. Hartsell and Russell W. Bunting as speakers. At the Wednesday evening General Session, Dr. Joseph C. Bloodgood, M. D., of the John Hopkins University, will discuss "The Early Recognition of Pre-cancerous Lesions of the Mouth and Tongue." At the Thursday evening General Session two selected papers will be presented from Sections I and III.

The program for the Section meetings has not been entirely completed, and two or three papers will be added to the following list: Dr. J. R. Callahan, Cincinnati, Some phase of Root Canal Treatment; Dr. W. H. DeFord, Des

Moines, Some phase of Eliminating Pain; Dr. E. J. Eisen, Milwaukee, "Dental Radiography;" Dr. Herbert L. Wheeler, New York City, subject to be announced; Dr. Fred. W. Gethro, Chicago, subject to be announced; Dr. J. D. Patterson, Kansas City, "Pyorrhea Alveolaris;" Dr. C. H. Oakman, Detroit, "Oral Hygiene;" Dr. Chalmers J. Lyons, Ann Arbor, "The Pathological Significance of Impacted Teeth;" Dr. Dayton Dunbar Campbell, Kansas City, "Some Basic Principles and Methods in the Reproduction of Mandibular Movements;" Dr. Wm. A. Giffin, Detroit, "Technique for Making Impressions and Models for the Construction of Artificial Dentures" Demonstrated with motion pictures; Dr. A. J. Bush, Columbus, "Classification of Fixed Bridgework with Law Governing its Application;" Dr. Carl B. Case, Milwaukee, "Evolution of Root Movement;" Dr. Jules J. Sarrazin, New Orleans, "Properly Constructed Bridges and their Hygienic Care;" Dr. Homer C. Brown, Columbus, "The Responsibilities of the State Society Officers;" Dr. Otto U. King, Huntington, "The Business Side of the State Society Work."

The Clinic Committee is to present a Progressive Clinic Wednesday morning commencing at 9:30. They have secured a list of exceptionally high class clinicians for both the Progressive and the General Clinic. The General Clinic will be given Friday morning and full details of the clinical program will be presented through the National Bulletin and later journals.

The Local Committee has selected the Powers Hotel as Headquarters and reservations should be made as early as possible. A full list of hotels and rates will appear in the National Bulletin. This Committee has made ample provisions for a large meeting. All except the evening General Sessions will be held at the Exposition Park under most favorable conditions. The Superintendent of the Park has assured us that the temperature of these buildings can be regulated so that July weather need not interfere with our comfort.

All reputable practitioners of Dentistry and Medicine are cordially invited to attend this meeting.

Fraternally,

Otto U. King, Gen. Sec'y. Huntington, Ind.

Homer C. Brown, President. Columbus, Ohio.

Railway Passenger Rates, to and from Rochester, N. Y.
(July 7th to 10th, 1914.)

1. The railways of the Trunk Line Assn. covering New York State (east of and including Buffalo, Niagara Falls and Salamaca), New Jersey, Pennsylvania (east of and including Erie, Oil City and Pittsburgh), Delaware, Maryland, District of Columbia, Virginia and West Virginia (east of and including Wheeling, Parkersburg and Huntington), have given an open rate of two cents per mile in each direction in their respective territories with the minimum excursion rate of $1.00.

Tickets to be sold and good going July 5th to 7th, 1914, and returning to reach original starting point not later than July 13th.

2. The New England Passenger Assn. covering the railways of New England also grant the above privileges and limitations with tickets from their principal stations. The agent at other stations will require not less than 48 hours notice to procure fares and tickets obtainable from the General Passenger Department of the railroad interested.

3. Eastern Canadian Passenger Assn.—Canada (east of and including Port Arthur, Sault Ste. Marie and St. Clair and Detroit Rivers), declined granting reduced fares.

4. Central Passenger Assn.—Territory west of Buffalo, Pittsburgh, Wheeling, Parkersburg and Huntington to and including Chicago, and St. Louis and north of the Ohio River, including Cincinnati, Louisville and Cairo, have granted a rate of two cents per mile, in each direction added to the tender received from Trunk Lines, through fares however, not to be higher than the 30 day summer tourist fares to Buffalo, N. Y. plus tender covered.

Signature form of tickets to be sold on July 4th, 5th, 6th, with return limit to reach starting point not later than midnight of July 14th, 1914, except in border territory common to the Trunk Lines, selling dates July 5th, 6th, 7th, with return limit of July 13th. Your committee suggests confering with local agent for excursion rate with longer limit, if desired.

5. Southeastern Passenger Assn—Territory south of Ohio and Potomac and east of Mississippi Rivers, declined granting a concession in rates and suggest that the summer excursion tickets will be on sale daily before time of meeting, from the principal stations in their territory, reaching Buffalo, Niagara Falls and other points contiguous to Rochester.

6. Western Passenger Assn—Territory west of Chicago, Peoria and St. Louis to and including Denver, Colo., and Cheyenne, Wyo., state that the summer tourist fares to Eastern sections will be available from principal points in their territory. The general basis of fares—two cents per mile in each direction to their Eastern gateways added to the fares over their lines.

Confer with local ticket agents.

7. Southwestern Passenger Assn—Territory southwest of St. Louis, including Texas, Arkansas, Oklahoma, Missouri (south of Missouri River), and Louisiana (west of Mississippi River), and Mexico, suggest that the summer excursion rates are practically two cents per mile in each direction. Tickets on sale daily May 15th to September 30th, limited to return October 31st.

Confer with local agents.

8. The territory covered by the Trans-Continental Passenger Assn. Pacific coast and other far western territory not otherwise covered by the above associations, suggest that the summer excursion rate $72.50 is as low as can be granted from San Francisco to Chicago and return. Sale dates for tickets June 29th, 30th, and July 2nd, 3rd.

Excursion tickets from Oregon and Washington to Chicago, daily during June and July.

9. Convenient trains to Rochester—

| | | | |
|---|---|---|---|
| Lv. St. Louis, Mo | Big Four Route | 11.30 | a.m. |
| Lv. Indianapolis, Ind | " | 5.50 | p.m. |
| Lv. Cincinnati, Ohio | " | 6.05 | p.m. |
| Lv. Dayton, Ohio | " | 7.45 | p.m. |
| Lv. Springfield, Ohio | " | 8.30 | p.m. |
| Lv. Columbus, Ohio | " | 9.55 | p.m. |
| Ar. Rochester, N. Y | N. Y. Central R. R | 9.21 | a.m. |

| | | | |
|---|---|---|---|
| Lv. Chicago, Ills | Lake Shore & Mich. So. Ry | 5.30 | p.m. |
| Lv. Toledo, Ohio | " | 11.15 | p.m. |
| Ar. Rochester, N. Y | N. Y. Central | 8.45 | a.m |

| | | | |
|---|---|---|---|
| Lv. Chicago, Ills | Michigan Central R. R | 5.40 | p.m. |
| Lv. Grand Rapids, Mich | " | 5.10 | p.m. |
| Ar. Rochester, N. Y | New York Central | 9.21 | a.m. |

Parlor Cars and Sleepers over these lines reach Rochester via N. Y. Central.

Through tickets to New York permit stop-over of ten days at Rochester by depositing ticket at Station Ticket Office immediately upon arrival. A convenience to those joining the European Tour or visiting the Metropolis.

10. From New York to Rochester—Excursion tickets sold July 5th, 7th, return by July 13th.

| | | |
|---|---|---|
| Lv. New York, N. Y | New York Central | 8.30 a.m.<br>9.34 p.m.<br>11.35 p.m. |
| Ar. Rochester, N. Y | " | 4.05 p.m.<br>6.30 a.m.<br>8.13 a.m. |

Rate to Rochester—excursion both directions $14.45

| | | |
|---|---|---|
| Lv. New York, N. Y | West Shore R. R | 8.35 a.m.<br>7.20 p.m. |
| Ar. Rochester, N. Y | " | 6.40 p.m.<br>5.12 a.m. |

Rate to Rochester—excursion both directions $13.40

| | | |
|---|---|---|
| Lv. New York, N. Y | Lehigh Valley R. R | 9.50 a.m.<br>11.50 a.m.<br>8.50 p.m. |
| Ar. Rochester, N. Y | " | 9.44 p.m.<br>9.44 p.m.<br>8.25 a.m. |

| | |
|---|---|
| Rate to Rochester—excursion both directions | $13.40 |
| For ten or more people, traveling on one ticket | $13.20 |
| Black Diamond Express (11.50 a.m.) fare | $13.40 |
| Pullman seat | $1.75 |

Your Committee suggests that members confer with local railway agents with reference to excursion rates to Rochester or nearby points, with stop-over privileges.

National Dental Association, Committee on Transportation: V. H. Jackson, Chairman, New York; H. F. Hoffman, Denver, Colo.; L. P. Dotterer, Charleston, S. C.; T. S. Smith, Palo Alto, Cal;. Wm. W. Belcher, Rochester, N. Y.

